# *Escherichia coli* and *Salmonella* Enteritidis dual-species biofilms: interspecies interactions and antibiofilm efficacy of phages

**DOI:** 10.1038/s41598-019-54847-y

**Published:** 2019-12-03

**Authors:** Catarina Milho, Maria Daniela Silva, Diana Alves, Hugo Oliveira, Clara Sousa, Lorenzo M. Pastrana, Joana Azeredo, Sanna Sillankorva

**Affiliations:** 1 Centre of Biological Engineering, LIBRO – Laboratório de Investigação em Biofilmes Rosário Oliveira, University of Minho, 4710-057 Braga, Portugal; 2 LAQV/REQUIMTE, Chemical Science Department, Faculty of Pharmacy, University of Porto, 4050-313 Porto, Portugal; 3 INL- International Iberian Nanotechnology Laboratory, Av. Mestre José Veiga, 4715-330 Braga, Portugal

**Keywords:** Biological techniques, Biotechnology, Ecology, Microbiology

## Abstract

*Escherichia coli* and *Salmonella* Enteritidis are foodborne pathogens forming challenging biofilms that contribute to their virulence, antimicrobial resistance, and survival on surfaces. Interspecies interactions occur between species in mixed biofilms promoting different outcomes to each species. Here we describe the interactions between *E. coli* and *S*. Enteritidis strains, and their control using specific phages. Single-species biofilms presented more cells compared to dual-species biofilms. The spatial organization of strains, observed by confocal microscopy, revealed similar arrangements in both single- and dual-species biofilms. The EPS matrix composition, assessed by Fourier-transform infrared spectroscopy, disclosed that the spectra extracted from the different dual-species biofilms can either be a combination of both species EPS matrix components or that the EPS matrix of one species predominates. Phages damaged more the single-species biofilms than the mixed biofilms, showing also that the killing efficacy was greatly dependent on the phage growth characteristics, bacterial growth parameters, and bacterial spatial distribution in biofilms. This combination of methodologies provides new knowledge of species-species and phage-host interactions in biofilms of these two major foodborne pathogens.

## Introduction

The presence of pathogenic and spoilage bacteria in food products is a known worldwide problem that not only leads to food spoilage but is linked to many foodborne outbreaks. In food industries, product contamination can occur at different food processing stages, via direct contamination and also cross-contamination^1^. Direct contamination of foods may occurs due to exposure to pathogens present in the soil, contaminated irrigation waters, and animals in the growing area^2^. Cross-contamination of the food products may be a result of moisture drops and aerosols, which indirectly contaminate working surfaces and products, due to contaminated washing waters, and improper handling of the products by the workers, for instance, due to poor hand sanitation^3^. *E. coli* and *Salmonella* are two major foodborne pathogens frequently isolated from varied surfaces, such as hard-to-reach areas that are not cleaned regularly (e.g. underside of conveyor belts, and pipelines)^4^, soil^5,6^, product washing waters^7^, and food products^8^. According to the US  Centers for Disease Control and Prevention (CDC), these two species are responsible for millions of illnesses, 2,000 and 23,000 hospitalizations, and 60 and 450 deaths every year in the US alone, respectively^9^.

Both *E. coli* and *Salmonella* have been found in the form of biofilms attached to varied surfaces. There, these species, secrete extracellular polymeric substances (EPS) that maintain the cells together forming complex 3D structures consisting mainly of proteins, eDNA, and polysaccharides, among other components^10^. Biofilm cells tolerate high levels of antimicrobial agents which makes them extremely challenging to remove^10^. Although *E. coli* and *Salmonella* form single-species biofilms, they also coexist in multispecies communities on food processing surfaces or food products^11^. The interactions in dual-species biofilms can be positive, negative or neutral for each species^12^. Biofilms formed by *E. coli* and *Salmonella* displayed enhanced resistance to quaternary ammonium chloride (QAC)-based sanitizer (Vanquish, Total Solutions, WI, USA) that is a one-step concentrated cleaner, broad-spectrum disinfectant/virucide, sanitizer, with an application on a hard, non-porous surface. Resistance to QAC was due to the EPS produced by *Salmonella*, which conferred protection to both species^4^. The interaction between these two species in biofilms formed on HEp-2 cells showed that *Salmonella* biofilms outgrew and displaced pre-formed *E. coli* biofilms^13^. In another study, a specific strain of *Salmonella* unable to form biofilms utilized the curli proteins cross-seeded by *E. coli* to enhance its adherence to dual-species biofilms^14^.

Phages, the natural predators of bacteria, have been extensively employed in the control of single-species biofilms of common bacteria present in the food industry^15–20^. However, the application of phages to mixed bacterial populations is scarce but the results, reported to date, have been encouraging. For instance, phages successfully controlled dual-species biofilms of *Staphylococcus lentus* and *Pseudomonas fluorescens*, and further were able to decrease the number of the non-susceptible strain, when its specific phage was not added, through their release from the biofilm consortia^21^. Dual-species biofilms of *Staphylococcus aureus* and *Staphylococcus epidermidis* challenged with phages phiIPLA-RODI and phiIPLA-C1C in a cocktail also reduced significantly the number of attached cells^22^.

The focus of this work was to assess the influence of *E. coli* and *S*. Enteritidis strains on single- and dual-species biofilm formation, and also to investigate biofilm control using phages vB_EcoM_Daica (Daica) and φ135, specific for *E. coli* and *S*. Enteritidis, respectively.

## Results

### Characterization of *E. coli* and *S*. Enteritidis strains growth parameters

The constructed *E. coli* and *S*. Enteritidis strains carrying sfGFP- and mCherry-expression plasmids were characterized to determine their specific growth rates and doubling times (Table 1). The differences in EC 434 and EC 515 growth, and of SE Ex2 and SE 269 resulted in statistically different µ_max_ values (p < 0.05) (Table 1). Furthermore, also the µ_max_ between EC 515 and SE 269 was statistically significant (p < 0.05) with EC 515 presenting the slowest doubling time (t_d_).

**Table 1:** Specific growth rate (µ_max_) and doubling time (t_d_) of the *E. coli* and *S*. Enteritidis strains used

Strains	µ_max ± _SD (h^−1^)	t_d_ ± SD (min)
EC 434	0.763 ± 0.03*	54.50 ± 2.44
EC 515	0.902 ± 0.094*^,†^	46.10 ± 4.83^†^
SE Ex2	0.791 ± 0.038*	52.57 ± 2.45*
SE 269	0.668 ± 0.073*^,†^	62.25 ± 12.50*^,†^

Statistical analysis of the results was performed using GraphPad Prism 6 (GraphPad Software, La Jolla, CA, USA). Differences between strains and species parameters were assessed using two-way ANOVA followed by Tukey’s multiple comparison statistical test Asterisks (*) indicate significant differences between strains (*p < 0.05) and daggers (^†^) indicate significant differences between species (p < 0.05).

### Characterization of single- and dual-species biofilms

**Figure 1:**
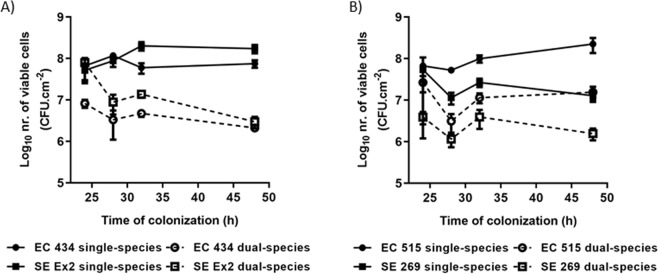
Biofilm formation of (**A**) EC 434 and SE Ex2 and (**B**) EC 515 and SE 269, single and dual-species. Comparison values of single- versus dual-species biofilms are all statistically significant (p < 0.05), except for EC 515 single- versus EC 515 + SE 269 dual-species biofilm, at 24 h of growth, and SE 269 single- versus SE 269 + EC 515 dual-species biofilm, at 24 h of growth.

The four strains were characterized according to a methodology described previously^23^ to find if these were weak or strong adherent bacteria. Species had differences in adherence and consequent biofilm formation and thus for the dual-species biofilms, two sets of combinations that were used consisting of the strong (EC 434 and SE Ex2) and the weak (EC 515 and SE 269) biofilm-formers. Overall, all strains presented higher numbers in single-species biofilms compared to dual-species biofilms (Fig. 1). For instance, biofilms of EC 434 (7.5 × 10^7^ CFU/cm^2^) and SE Ex2 (1.73 × 10^8^ CFU/cm^2^) dropped to approximately 2.5 × 10^6^ CFU/cm^2^ when added together (Fig. 1A). Also, EC 515 and SE 269 biofilms showed 1 log less viable cells in dual-species biofilms compared to single-species biofilms (Fig. 1B).

**Figure 2:**
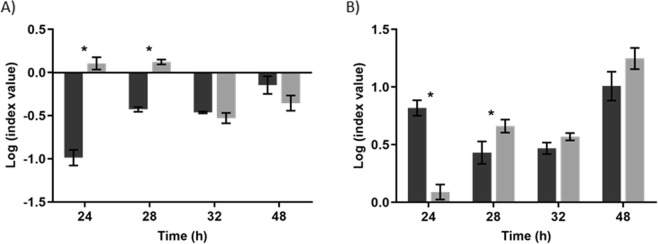
Relative Increase Ratio (RIR) and Competitive Index (CI) obtained for (**A**) EC 434 alone and when combined with SE Ex2, and (**B**) EC 515 alone and when combined with SE 269, respectively. Statistical analysis of the results was performed using GraphPad Prism 6 (GraphPad Software, La Jolla, CA, USA). Differences between single- and dual-species biofilm formation abilities were assessed using two-way ANOVA followed by Tukey’s multiple comparison statistical test. Asterisks (*) indicate a significant difference (p < 0.05) between CI and RIR.

To better understand the differences between the *E. coli* and *S*. Enteritidis strains and their biofilm-forming abilities, the Competitive Index (CI) and the Relative Increase Ratio (RIR) indexes were calculated (Fig. 2). While in CI the growth curves of the two species in mixed biofilms are compared, the RIR compares the growth curves of both species in single-species biofilms. A negative CI was detected throughout all time points (Fig. 2A). However, only at 24 and 28 h of biofilm growth of SE Ex2 + EC 434 the CI and RIR were found to be statistically different (p < 0.05), indicating a competitive advantage of SE Ex2 over EC 434. On the other hand, a positive CI was observed at 24 and 28 h of growth for EC 515 + SE 269 biofilms, indicating a competitive advantage for EC 515 (Fig. 2B).

**Figure 3:**
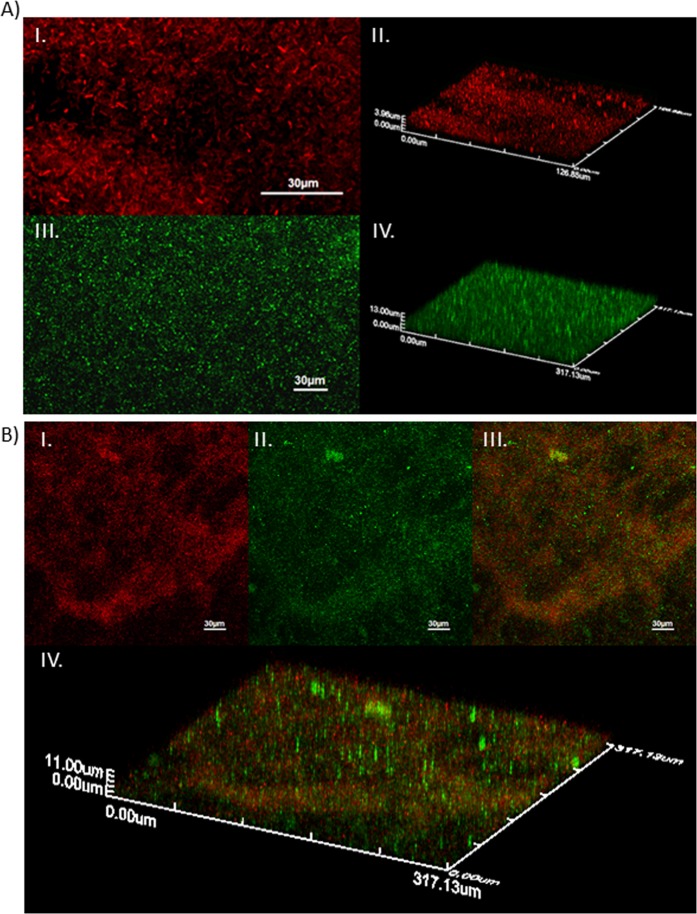
CLSM images showing spatial organization of (**A**) EC 434 (I – 2D, II – 3D) and SE Ex2 (III – 2D, IV – 3D) single-species biofilms, and (**B**) EC 434 + SE Ex2 dual-species biofilm (I – EC 434 colored in red; II – SE Ex2 colored in green; III – superposition of both colors, 2D; IV – biofilm 3D spatial distribution).

**Figure 4:**
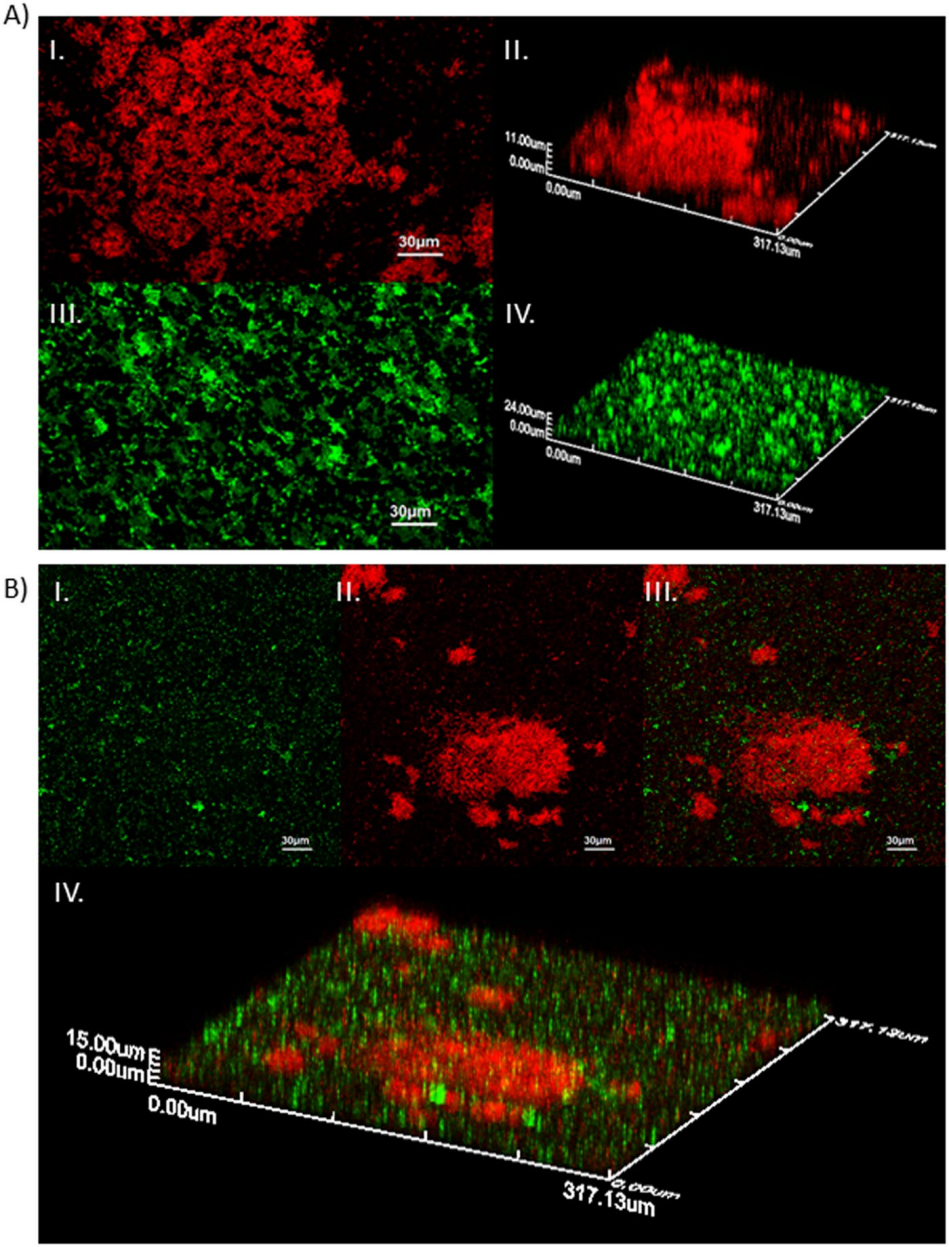
CLSM images showing spatial organization of (**A**) EC 515 (I – 2D, II – 3D) and SE 269 (III – 2D, IV – 3D) single-species biofilms, and (**B**) EC 515 + SE 269 dual-species biofilm (I – SE 269 colored in green; II – EC 515 colored in red; III – superposition of both colors, 2D; IV – biofilm 3D spatial distribution).

The bacterial distribution within the 48-h-old single- and dual-species biofilms formed by *E. coli* strains (mCherry fluorophore), and/or *S*. Enteritidis (sfGFP) was visualized using CLSM (Figs. 3 and 4). EC 434 single-species biofilms presented a heterogeneous spatial distribution, with bacteria accumulated more in some areas than others, reaching a thickness of approximately 4 µm (Fig. 3A, I and II). SE Ex2 biofilms were more evenly spread throughout the polystyrene coupon, presenting a thickness of ≈13 µm (Fig. 3A, III and IV). The difference in thickness between EC 434 and SE Ex2 biofilms was is in agreement with the number of viable cells obtained for each biofilm at 48 h (Fig. 1A). Analysis by CLSM of dual-species biofilm formed by EC 434 + SE Ex2 showed both strains growing on the same areas on the coupon, suggesting an influence of each species on the spatial distribution of the other species (Fig. 3B, I–III). The thickness EC 434 + SE Ex2 biofilms reached approximately 11 µm (Fig. 3B, IV), due to the lower number of bacterial counts obtained (1.55 Log_10_ CFU/cm^2^ and 1.76 Log_10_ CFU/cm^2^ for EC 434 and SE Ex2) (Fig. 1A).

The spatial characterization was also assessed for 48-h single- and dual-species biofilms of EC 515 and SE 269 (Fig. 4). EC 515 alone formed clustered biofilm structures, reaching, in some areas of the coupon, 11 µm in thickness (Fig. 4A, I and II). EC 434 strain reached only a  4 µm thickness (Fig. 3A, I and II). SE 269 formed a biofilm with a reticulate appearance equally dispersed through the coupon with a thickness of approximately 24 µm (Fig. 4A, III and IV), while SE Ex2 presented only a 13 µm thickness. EC 515 + SE 269 biofilms, maintained their single-species biofilm spatial arrangement (Fig. 4B, I–III) although reaching only a maximum thickness of ≈15 µm (Fig. 4B, IV).

**Figure 5:**
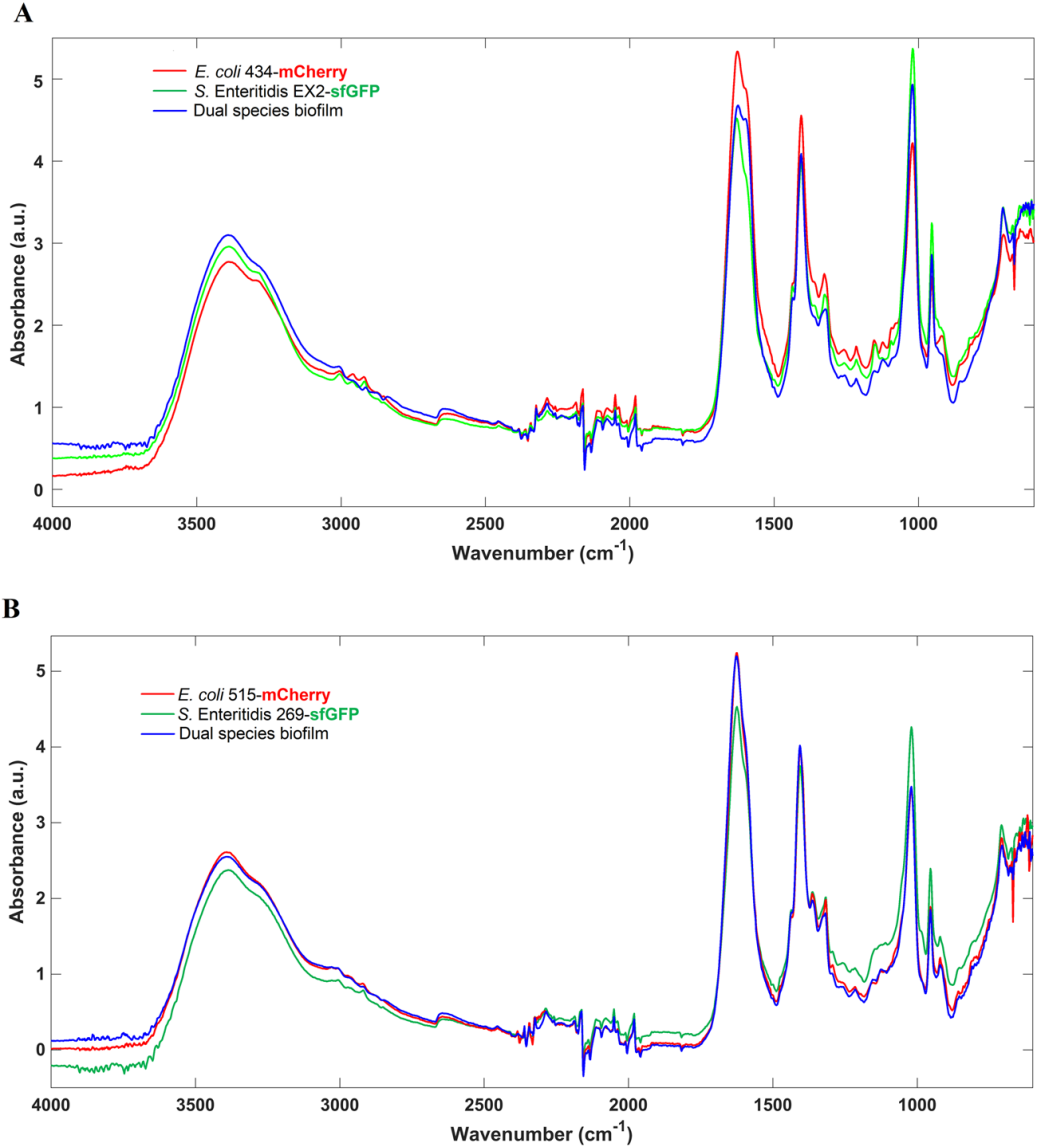
Mean infrared spectra, processed with standard normal variate, of the EPS extracted from single- and dual-species biofilms of (**A**) EC 434 + SE Ex2 and (**B**) EC 515 + SE 269. Peaks: 1 – lipids, 2 - proteins/amides I and II, 3 - phospholipids and nucleic acids, 4 – polysaccharides.

**Figure 6:**
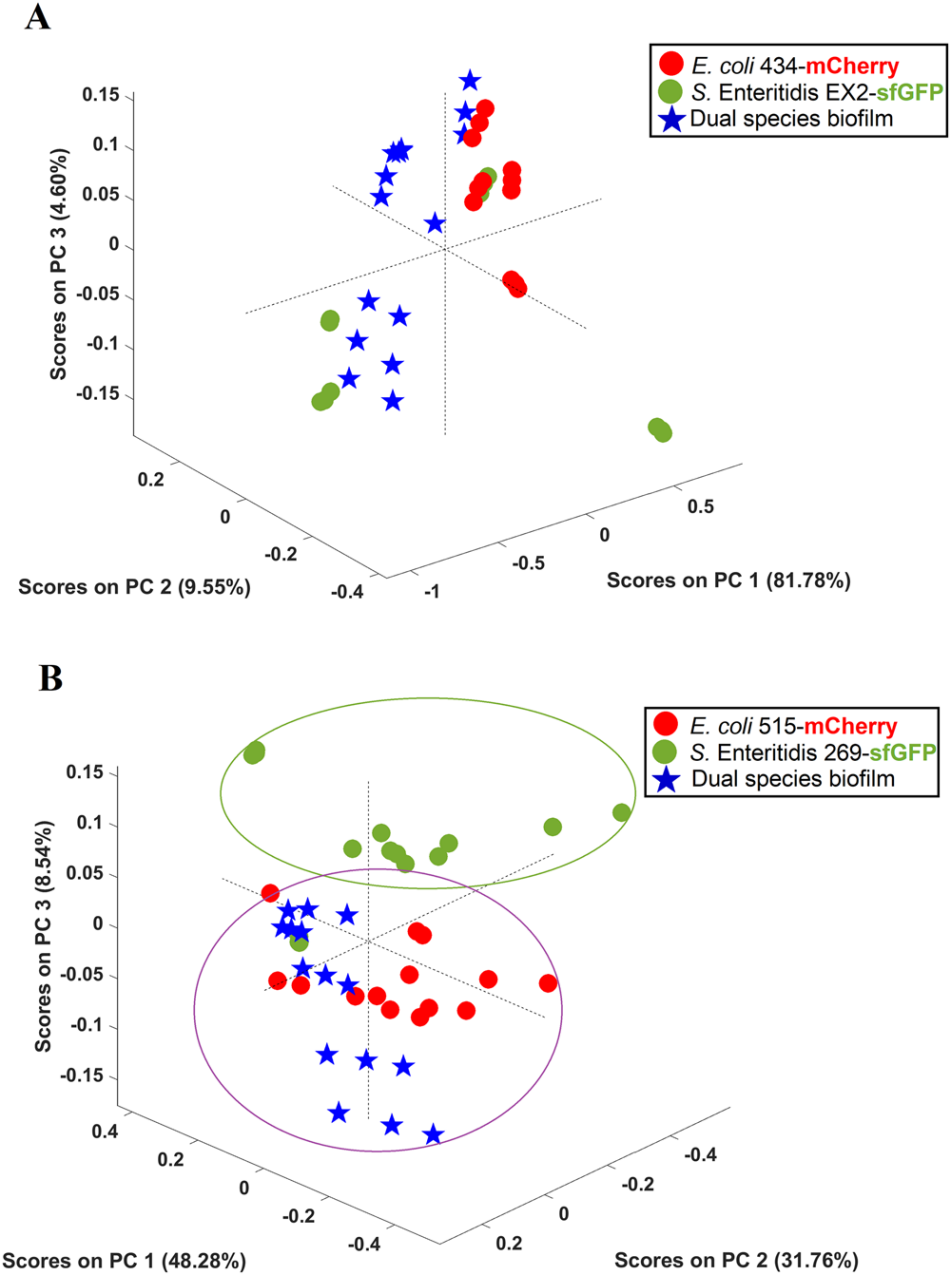
Scores map of the PCA model of the EPS infrared spectra extracted from single- and dual-species biofilms of strong (**A**) and weak (**B**) producers. The PCA model was built considering the spectral regions from 3600–2800 cm^−1^ and 1700–900 cm^−1^.

EPS spectra obtained by FTIR-ATR spectroscopy of *E. coli* and *S*. Enteritidis from single- and dual-species biofilms was analyzed (Fig. 5). Globally, the spectra presented a very similar and typical shape containing the absorption bands of lipids (3000–2800 cm^−1^), proteins/amides I and II (1700–1500 cm^−1^), a mixed region of phospholipids and nucleic acids (1500–1185 cm^−1^), polysaccharides (1185–900 cm^−1^), and the fingerprint region (900–600 cm^−1^)^24,25^. Regarding the EC 434 + SE Ex2 mixed biofilm EPS matrix, the spectrum is similar to EC 434 or to SE Ex2 spectra, depending on the considered spectral region (Fig. 5A), pointing to the absence of a predominant EPS matrix species. Additionally, the PCA model revealed an undefined clusterization with all spectra being quite dispersed in the whole scores map (Fig. 6A). Concerning the EC 515 + SE 269 EPS matrix, this spectrum is more similar to the one of EC 515. Indeed, the infrared spectra of the dual and single-species biofilms are almost totally superimposable with the EC 515 EPS matrix, being quite different from the SE 269 spectrum (Fig. 5B). The corresponding scores map of the PCA model also reflects this similarity, showing two defined clusters, one containing the EPS spectra from *S*. Enteritidis biofilm and the other containing the EPS matrix spectra from both single *E. coli* and the dual-species biofilms (Fig. 6B).

### Phage characterization

**Figure 7:**
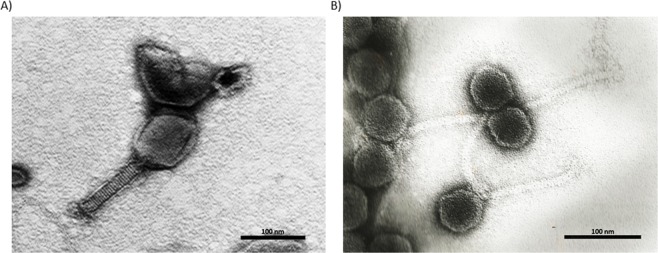
TEM micrographs of *E. coli* phage Daica (**A**) and *S*. Enteritidis phage φ135 (**B**). The scale bar is 100 nm.

Transmission electron microscopy (TEM) visualization showed that *E. coli* phage Daica is a myovirus, having a long contractile tail (Fig. 7A), while the *S*. Enteritidis phage φ135 is a siphovirus with a long non-contractile tail (Fig. 7B).

**Figure 8:**
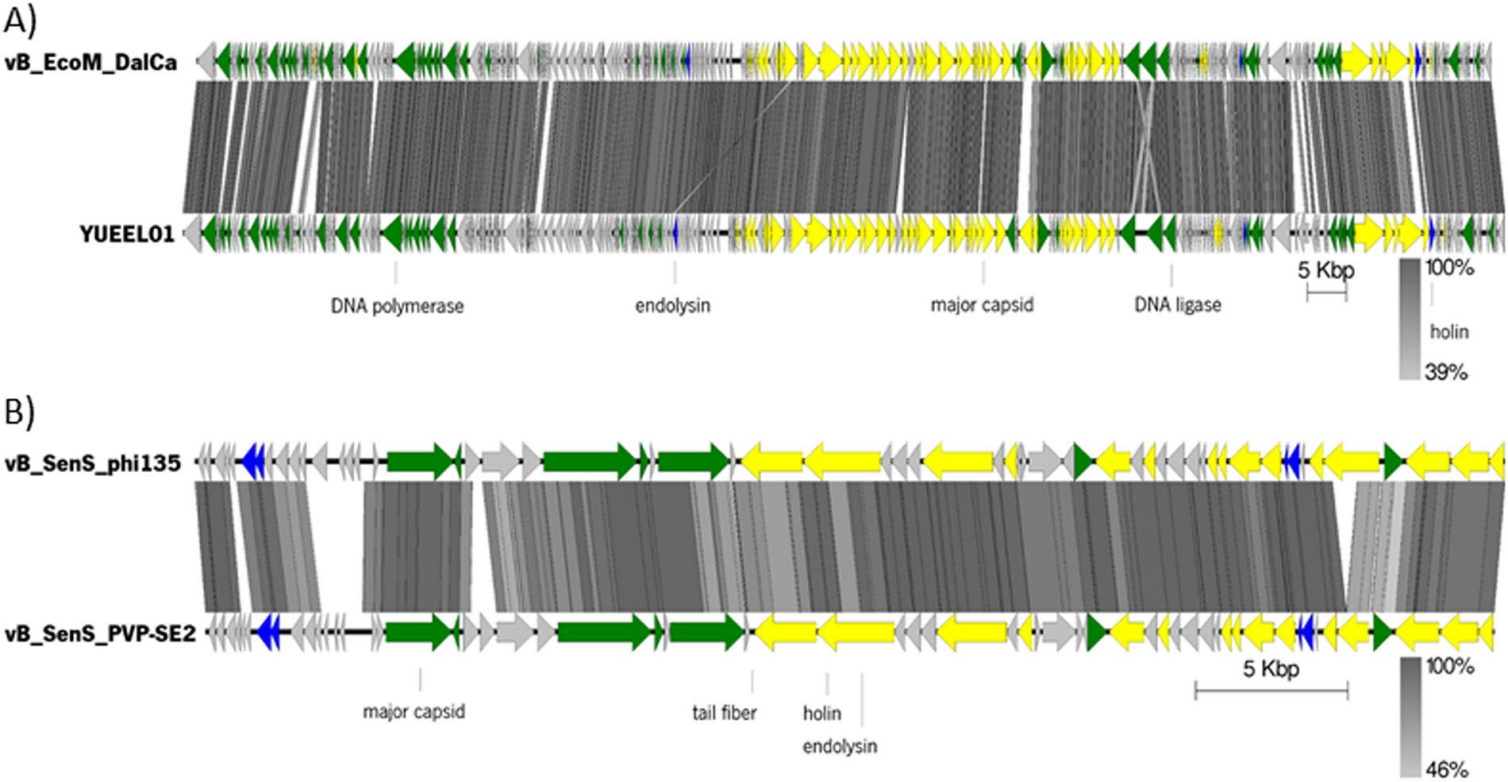
Linear map of phages (**A**) Daica and (**B**) φ135 genome sequences. The arrows point the direction of transcription, represent the predicted ORFs and are colored (yellow, green, blue, and grey) according to their predicted functions. Major transcriptional units are represented. Schematic representation of the genomic organization of phage Daica compared to *E. coli* phage YUEEL01 and phage φ135 to *S*. Enteritidis phage vB_SenS_PVP-SE2 using Easyfig.

Genomic analysis of Daica revealed a genome of 166,040 bp in length, encoding 268 putative CDSs (130 with known function), regulated with 4 bacterial promoters and 34 rho-independent terminators. Whole-genome comparison showed *Escherichia* phage YUEEL01 (KY290975) as the closest homolog, with which it shares 94.1% of nucleotide identity and 256 genes (Fig. 8A). Regarding φ135, the genome is 43,142 bp in length, encodes 59 CDSs (28 with a predicted function), 7 bacterial promoters and 22 rho-independent terminators. Most proteins have high homology (>90% amino acid identity) to *S*. Enteritidis phage PVP-SE2 (MF431252) proteome. Genomic comparisons show that φ135 is collinear with vB_SenS_PVP-SE2 phage, sharing 91% nucleotide identity and 53 genes (Fig. 8B).

**Figure 9:**
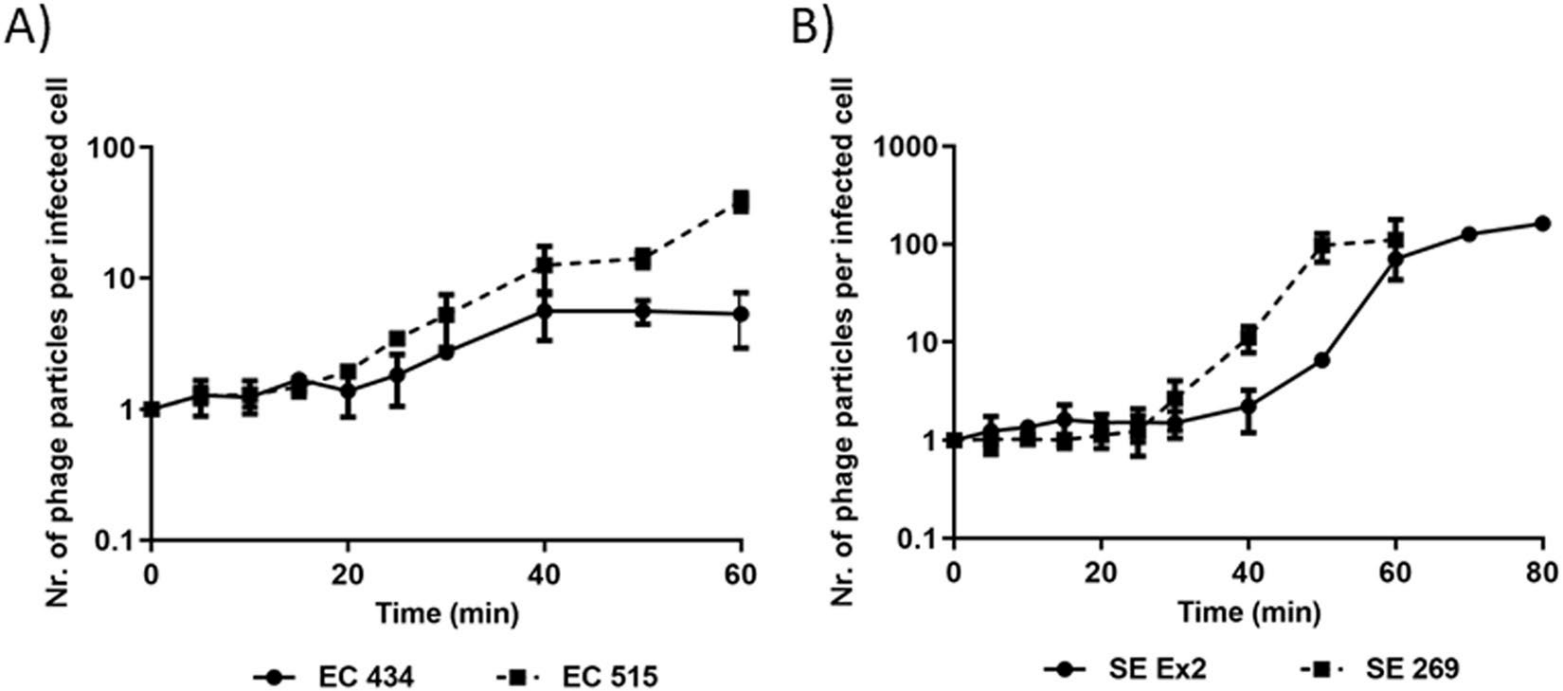
One-step growth curves of phage Daica on EC 434 and EC 515 (**A**), and phage φ135 on SE Ex2 and SE 269 (**B**), at room temperature.

One-step growth curves of Daica and φ135 phages revealed that Daica presented a 20 min latent period on EC 434 and 15 min on EC 515, respectively (Fig. 9). Furthermore, Daica produced only 5.3 PFU per infected cell on EC 434, and 38.9 PFU per infected cell on EC 515 (Fig. 9A). Phage φ135 had a latent period of approximately 25 min on SE 269, and 30 min on SE Ex2 giving origin to 110.9 and 162.9 particles per infected SE 269 and SE Ex2 cell (Fig. 9B).

### Phage treatment of single- and dual-species *E. coli* and *S*. Enteritidis biofilms

**Figure 10:**
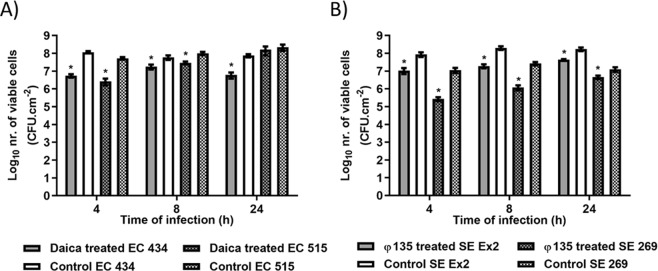
*E. coli* (**A**) and *S*. Enteritidis (**B**) single-species biofilms formed for 24 h in 96-well plates at 37 °C treated with phages Daica and φ135, respectively, for 4, 8 and 24 h. (**A**) Solid bars correspond to EC 434 biofilms treated with Daica (grey) or untreated/control (white) while shaded bars correspond to the control EC515 (white with black squares) or EC 515 treated with Daica (grey with black squares). (**B**) Solid bars correspond to SE Ex2 biofilms treated with φ135 (grey) or untreated/control (white) while shaded bars correspond to the control SE Ex2 (white with black squares) or SE 269 treated with φ135 (grey with black squares). Statistical analysis of the results was performed using GraphPad Prism 6 (GraphPad Software, La Jolla, CA, USA) using two-way ANOVA followed by Tukey’s multiple comparison statistical test. Asterisks (*) indicate a significant difference (p < 0.05) between phage-treated and control samples.

Phages were used on 24 h single-species biofilms formed by EC 434 and EC 515 (phage Daica), and SE Ex2 and SE 269 (phage φ135) (Fig. 10). Daica produced the highest reduction in the number of biofilm viable cells at 4 h of treatment for both strains [EC 434 (1.33 Log_10_), and EC 515 (1.29 Log_10_)] (Fig. 10A). SE Ex2 and SE 269 biofilms challenged with φ135 showed a greater bacterial reduction at 8 h for SE Ex2 biofilms (1.02 Log_10_), and at 4 h for SE 269 (1.63 Log_10_) (Fig. 10B).

**Figure 11:**
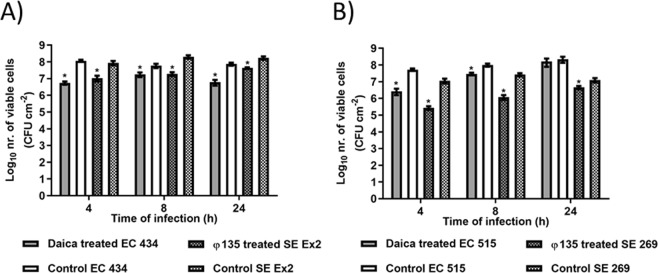
EC 434 + SE Ex2 (**A**) and EC 515 + SE 269 (**B**) dual-species biofilms formed for 24 h in 96-well plates at 37 °C treated with phages Daica and φ135 for 4, 8 and 24 h. (**A**) Solid bars correspond to EC 434 biofilms treated with Daica (grey) or untreated/control (white) while shaded bars correspond to the control SE Ex2 (white with black squares) and SE Ex2 treated with φ135 (grey with black squares). (**B**) Solid bars correspond to EC 515 biofilms treated with Daica (grey) or untreated/control (white) while shaded bars correspond to the control SE 269 (white with black squares) or SE 269 treated with φ135 (grey with black squares). Statistical analysis of the results was performed using GraphPad Prism 6 (GraphPad Software, La Jolla, CA, USA) using two-way ANOVA followed by Tukey’s multiple comparison statistical test. Asterisks (*) indicate a significant difference (p < 0.05) between phage-treated and control samples.

*E. coli* and *S*. Enteritidis dual-species biofilm control with *E. coli* phage Daica and *S*. Enteritidis phage φ135 was investigated (Fig. 11). Dual-species biofilms of EC 434 + SE Ex2 using the phage cocktail, resulted in a maximum viable cell decrease at 8 h, with EC 434 being reduced by 1.15 Log_10_ and SE Ex2 by 0.88 Log_10_ (Fig. 11A). EC 515 + SE 269 dual-species biofilms reached the lowest numbers of viable cells at 4 h of treatment having the phages reduced EC 515 by 1.07 Log_10_ and SE 269 at 8 h by 2.42 Log_10_ (Fig. 11B).

## Discussion

*E. coli* and *Salmonella* represent an important public health concern^26,27^, being responsible for numerous foodborne outbreaks, and often isolated from contaminated meats^28,29^, vegetables^30,31^, as well as food processing surfaces. Their presence in food processing surfaces leads often to food products cross-contamination events^32^. *E. coli* and *Salmonella*’s ability to form biofilms make them less susceptible to common disinfectants, physical removal, and other elimination processes^33,34^. In this work, we assessed the efficacy of two phages alone or in a cocktail, for the control of single- and dual-species biofilms, respectively. Furthermore, structural and compositional characteristics of *E. coli* and *S*. Enteritidis single-species biofilms were analysed, and dual-species biofilm interactions studied^35^.

The *E. coli* strains tested belong to serotypes O6 (EC 434) and O1:K1:H7 (EC 515). Mono-species biofilm formation by *E. coli* serotypes has been shown to be serotype-dependent^36^ but also influenced by the levels of curli expression, and presence/absence of virulence factors, such as Shiga toxins 1 and 2 (*stx1* and *stx2*), intimin (*eaeA*), enterohemolysin A (*hlyA*), fimbriae, and autotransporters (e.g. EhaB and EspP)^37–39^. For instance, a study with 39 Shiga toxin-producing *E. coli* isolates (seropathotypes A, B, and C), showed that seropathotype A, that includes the serotypes responsible for the highest incidence of foodborne outbreaks (O157:H7 and O157:NM), had greater ability to form biofilms than isolates from seropathotypes B (eg O26:H11, O45:H2, and O103:H2) and C (O157:H26)^38^. According to our results, significant discrepancies between the biofilm-forming ability by the *E. coli* serotypes O1 and O6 were perceived, with higher counts (1.5 Log_10_) in biofilms of EC 515 compared to EC 434 (Fig. 1). SE Ex2 and SE 269 isolates belong to *S*. Enteritidis serovar however their genotype is unknown. Thus assumptions equivalent to those described for *E. coli* cannot be established. The adherence ability after 24 h of SE Ex2 and SE 269 showed to be different, as also confirmed by the biofilm thickness using CLSM (Figs. 3 and 4). The CFU/cm^2^ determinations showed a 1.2 Log_10_ difference with SE Ex2 reaching higher numbers than SE 269 (Fig. 1). Single-species biofilms of *E. coli* treated with Daica resulted in equivalent antibiofilm efficacy against both strains used (Fig. 10). This result is possibly due to a combination of three different factors: the latent period of Daica in the tested strains and the burst size reached per infected bacterium (Fig. 9A); and also the strain growth parameters. Significant differences of µ_max_ were detected for both *E. coli* (EC 434 and EC 515) and *S*. Enteritidis (SE Ex2 and SE 269) (Table 1). Daica has a longer latent period and a lower burst size on EC 434 cell, compared to EC 515. Additionally, EC 434 strain has a higher doubling time which could result in less new cells to be infected by progeny phages. In EC 515, Daica produced around 7.3 times more progeny phage particles per infected cell, however, in this strain, the phage latent period was longer. All these factors influenced in the treatment outcome, having EC 515 treatment with Daica reached similar reductions (see Figs. 9A and 10A, and also Table 1). *Salmonella* phage φ135 showed a higher antibiofilm outcome in SE 269, compared to SE Ex2 biofilms. This is greatly due to a higher burst size in the first strain (Fig. 9). Also, the growth rate of SE 269 was lower and therefore the non-infected biofilm cells proliferated at a slower rate than the SE Ex2 non-infected cells, consequently resulting in a smaller number of descendent cells present at the sampling time points assessed (Figs. 9B and 10B, and also Table 1).

The strain combinations for the dual-species biofilm formation studies were: EC 434 + SE Ex2 (strong biofilm-formers), and EC 515 + SE 269 (weak biofilm-formers). The competitive ability of strains in mixed biofilms has been linked to the relative density of each bacterium added to form biofilms^40^, which in our study was exactly the same (200 µL/well at 1 × 10^7^ CFU/mL). Therefore, this factor cannot explain the higher/lower surface coverage by the different dual-species biofilms. The number of viable biofilm cells per area analysed in dual-species biofilms was always lower than in single-species biofilms (Fig. 1). Antagonism between biofilms formed with *E. coli* O157:H7 strain USDA 5 and *Salmonella* strain 457–88 was hypothesized to be due to species competition for adherence to the surface, leaving a smaller area available for adhesion and consequent biofilm formation by the less competitive species involved^41^. Mixed biofilm populations can also have a dominance of one species over the other. For instance, biofilms formed by *Staphylococcus aureus* and *E. coli* showed a dominance of the latter species, due to the shorter generation time of *E. coli* compared to *S. aureus*^42^. It seems that faster-growing bacteria control the environment, and favor their growth over other slower-growing strains resulting in a long-term prevalence in the surface area^43,44^. Growth characteristics between the two sets of species used for dual-species biofilms showed that only the set of strains EC 515 and SE 269 was significantly different (p < 0.05). This may explain why EC 515, presenting a faster growth rate, also covered a larger biofilm surface area when formed along with SE 269 (Fig. 1B). The EC 515 covered the surface area more in height forming preferentially 3D structures, while SE 269 cells were more scattered in the polystyrene surface (Fig. 4B). The same spatial distribution found in single-species biofilms was maintained by SE 269 and EC 515 when grown together.

The other set of strains used in dual-species biofilms, EC 434 and SE Ex2, had similar µ_max_ and t_d_ values (p > 0.05) being present in the biofilms in fairly similar numbers (Fig. 1A) and equally distributed in the same areas of the coupons (Fig. 3B).

FTIR-ATR spectroscopy coupled with chemometrics, a reliable and alternative method to accurately discriminate bacteria at different taxonomic levels, including *E. coli* clones^45^, and *Salmonella* serogroups and serotypes^46^, and has also been used in biofilm contexts. For instance, FTIR-ATR has been used to monitor biofilm *in situ*, in real-time, and under fully hydrated conditions^47^, the early stages of biofilm formation, and the EPS matrix from biofilms at mid-development and mature phases^47^. In the latter work, FTIR-ATR spectroscopy showed that mature biofilms presented substantially higher protein and carbohydrate peaks, and a low level of lipids that was, nonetheless, higher than in mid-development phase biofilms^47^. In the present work, FTIR-ATR was used to compare the EPS matrix composition of single- and dual-species biofilms (Fig. 5). The FTIR-ATR analysis showed few spectral differences among the samples with a lower absorption band of lipids in all EPS samples analyzed than the absorption bands of proteins or carbohydrates. Also, it seemed that the ratio between proteins/carbohydrates was higher for EC 515 and SE 269 (Fig. 5B). A previous study has reported a competitive advantage of EPS producer strains in mixed biofilm populations and also reported that this EPS secretion helps progeny cell movement up and out of the focal cell layer, providing better oxygen conditions to their descendants^48^. Taking into account the EPS matrix spectral profiles obtained by FTIR-ATR, the mixed biofilms of EC 434 + SE Ex2 resembled the EPS spectra obtained with EC 434 in terms of proteins/amides I and II, and the phospholipids and nucleic acids absorption peaks, and with the polysaccharide absorption band of SE Ex2 (Fig. 5A). So, in general, in this set of strains, none seemed to be a better EPS producer than the other. Although some differences in spectral band intensities were observed, FTIR-ATR spectroscopy is a semi-quantitative method and therefore quantitative relations about biofilm matrix components should be taken carefully. The polysaccharide component of the EPS matrix offers diverse benefits to the cells in biofilms, sustaining surface and cell-to-cell adhesions, providing protection, and allowing cell growth in 3D structures^49^. If we take into account only the polysaccharides absorption peak (peak 4), this clearly resembles the one obtained by SE Ex2 which suggests that this strain secretes more polysaccharides than EC 434. As described above, this can confer this strain a competitive advantage for surface adhesion and predominance in the mixed consortia (Fig. 5A) which is in agreement with the higher levels of SE Ex2 cells in the surface compared to EC 434 (see Fig. 1A and negative CI index in Fig. 2A). The other set of strains, EC 515 + SE 269, showed a FTIR-ATR spectra superimposing the spectrum of EC 515 observed in single-species biofilms (Fig. 5B). Therefore, it is clear that there is a predominance of the EC 515 EPS matrix in these dual-species biofilms but that also EC 515 predominated due to its µ_max_ (Table 1). Dominance of a surface by one specific bacterium in mixed biofilms can be growth rate-dependent or polysaccharide-dependent. However, this latter observation needs to be further validated, since only the FTIR-ATR absorbance peak between 1185 and 900 cm^−1^ was taken into consideration for this assumption.

The use of both phages as antibiofilm agents showed that short treatment periods are more effective towards both single- and dual-species biofilms (Figs. 10 and 11). The antibiofilm effect using the phage cocktail comprising Daica and φ135 phages, on dual-species biofilms formed by EC 434 and SE Ex2 resulted in similar Log_10_ reductions for both species (Fig. 11A). Both strains have non-statistically different µ_max_ and, therefore, in theory, the phage producing a higher burst size should reduce more biofilm cells. This was not, however, the situation observed after phage treatment of these biofilms. Taking into consideration that EC 434 and SE Ex2 biofilms also presented fairly similar viable cell numbers throughout the phage treatment experiments, and being φ135 able to produce 30.7 times more new phage particles per infected SE Ex2 cell compared to Daica on EC 434, this indeed should have resulted in greater SE Ex2 reductions. CLSM analysis showed that these strains are scattered throughout the coupon surface, and this can hinder phages from reaching their hosts (Fig. 3B). Furthermore, FTIR-ATR analysis of the EPS matrix also showed that the spectral peaks of these strains were more similar, in some peaks, to SE Ex2 (polysaccharide absorption peak, peak 4), and in other peaks more related to EC 434 (Fig. 5A). If the assumption made before, that SE Ex2 secretes more polysaccharides, this SE Ex2 EPS matrix can be masking the phage receptors present in the host cell surfaces of EC 434 explaining in this case, why a higher phage burst size did not result in an enhanced antibiofilm activity against EC 434. Nevertheless, further experiments need to be performed to understand this behavior, possibly resorting to time-lapse microscopy imaging, flow cytometry or even spinning disk confocal microscopy.

Phage challenge of the EC 515 and SE 269 dual-species consortia resulted in higher reductions of SE 269 biofilm cells than of EC 515 (Fig. 11B). In this case, this result is a reflection of φ135 having a better burst size than Daica, and also due to the slower growth rate of SE 269 (Fig. 10B and Table 1). Furthermore, CLSM showed that EC 515 preferentially formed 3D clusters, which can impair phage diffusion to reach cells that are in deeper biofilm layers (Fig. 4B). According to FTIR-ATR spectroscopy analysis, the EC 515 + SE 269 EPS spectrum obtained was remarkably similar to the spectrum of EC 515 EPS. These results indicate that, even though EC 515’s EPS predominates in the dual-species biofilm samples, it is possibly confined only to the clustered areas, and therefore does not constitute an obstacle for phage φ135 to reach SE 269 cells.

In conclusion, this work gives new insights into the many factors influencing dual-species biofilm development. Moreover, it sheds light on the interaction of single and cocktail phages for the control of single- and dual-species biofilms. A better understanding of species-species and phage-host interactions was reached through analysis of bacterial and phage growth parameters, viable cell determination, before and after phage treatment, CLSM imaging of the biofilm structures, and also EPS matrix analyses by FTIR-ATR spectroscopy. Even though some of the strains showed to be more competitive regarding their surface attachment, reaching higher viable cell numbers than the other species involved, this did not have a direct influence on the action of phages. Infectivity of the phage was more associated with their growth characteristics, in particular, the latent period duration and burst size, and also to the difficulty of phages to infect one particular strain, EC 515, which formed 3D biofilms structures rather than being uniformly dispersed over the surface, possibly impairing phage diffusion. Furthermore, the EPS matrix of one species can constitute a problem to the other one, and the phage killing ability the latter species, by possibly covering the phage receptors necessary for the adsorption step to take place.

## Materials and Methods

### Bacterial strains and phages

*E. coli* CECT 434 (EC 434, serotype O6 and Biotype 1) and CECT 515 (EC 515, serovar O1:K1(L1):H7), from the Spanish Type Culture Collection. The CECT 434 reference strain is recommended for use for in UNE-CEN ISO/TS 11133, EN ISO 6888, and ISO 15214 applications, and the CECT 515 for UNE-CEN ISO/TS 11133. The *S* Enteritidis Ex2 (SE Ex2) and 269 (SE 269) were previously isolated from contaminated food products (SE 269)^50^. All strains were grown at 37 °C in liquid or in solid LB medium (LB + 1.5% (w/v) of agar). The *Salmonella* phage used was φ135^50^, and the *E. coli* phage Daica was isolated, during this study, as previously described^51^.

### *E. coli* and *S*. Enteritidis electrocompetent cells

Electrocompetent cells were prepared by inoculating 100 mL of LB with 100 µL of overnight culture and incubated at 37 °C (200 rpm, Orbital Shaker ES-20/60, 10 mm orbit, BIOSAN, Latvia) until an OD_620_ ≈ 0.6. After, cells were chilled 20 min on ice, harvested (7,000 × *g*, 10 min), and the supernatant discarded. The pellet was sequentially washed with 100 mL of ice-cold dH_2_O; 50 mL, 25 mL and 10 mL of ice-cold glycerol (10% (v/v)), suspended in 1 mL of ice-cold glycerol (10% (v/v)), and stored as 100 µL aliquots at −80 °C until use.

### Construction of sfGFP and mCherry strains

*S*. Enteritidis strains were transformed with the sfGFP-pBAD plasmid (Addgene plasmid #54519) which encodes for the superfolder green fluorescent protein (sfGFP), and the *E. coli* strains with plasmid pNUT086, which encodes for the mCherry fluorophore. Electrocompetent *S*. Enteritidis and *E. coli* cells (100 µL) were mixed with the respective plasmids, placed in a 1 mm gap electroporation cuvette, and a pulse of 25 μF, 200 Ω, 1.8 kV was applied. After, 900 µL of liquid LB medium were added to the mixture, cells incubated at 37 °C (120 rpm, 1 h), and plated on solid LB supplemented either with ampicillin (100 µg/mL) or with kanamycin (50 µg/mL), for *S*. Enteritidis cells and *E. coli* cells, respectively.

### Characterization of the growth parameters of *E. coli* and *S*. Enteritidis strains

Specific growth rates and doubling times of all strains were determined according to previously described methods^52^. Experiments were performed in triplicate.

### Biofilm formation assessment by Crystal Violet staining

The method described by Stepanović *et al*. (2000) was used to categorize the biofilm-forming capacity of bacteria into: 1) non-biofilm-formers (OD ≤ ODc); 2) weak (OD_c_ < OD ≤ 2 × OD_c_); 3) moderate (2 × OD_c_ < OD ≤ 4 × OD_c_); and 4) strong biofilm-formers (OD > 4 × OD_c_). OD_c_ is the cut-off OD (ODc = OD average of sterile LB + 3 × Standard Deviation (SD) of the ODs of negative controls). Sterile LB served as negative control giving an average value of 0.089 ± 0.005 and an ODc of 0.104. Experiments were performed in triplicate.

### Biofilm formation

The ability of the different strains to form biofilms was determined according to a method using the microtiter plate biofilm model previously described^23^, with some modifications. Briefly, an overnight culture was grown (37 °C, 120 rpm) in LB supplemented with kanamycin or ampicillin, according to the plasmid used. After, all cultures were diluted to 1 × 10^7^ CFU/mL, and 200 μL/well of culture transferred to polystyrene microtiter plates (96-well flat-bottom, Sarstedt, Inc., Germany) and incubated at 37 °C (48 h, 120 rpm). After, biofilms were washed twice with saline and detached using an ultrasonic bath (50 kHz, 6 min, Sonicor SC-52, Sonicor Instruments, UK)^53^. Bacterial suspensions were homogenized, 10-fold serially diluted, and plated onto solid LB supplemented with either kanamycin or ampicillin, and incubated overnight at 37 °C. Experiments were performed in triplicate.

### Determination of the competitive index (CI) and the relative increase ratio (RIR)

In dual-species biofilms, the Competitive Index (CI) was established as the EC 434/SE Ex2 or EC 515/SE 269 ratios within the output sample divided by the corresponding ratio in the inoculum (input): CI = (EC 434/SE Ex2 or EC 515/SE 269) output/(EC 434/SE Ex2 or EC 515/SE 269) input, where output and input are the counts of viable cells [Log_10_(CFU/cm^2^)] obtained at defined time points or the inoculum (t = 0), respectively^12^. CI values were interpreted as follows: a CI value equal to 0 indicates equal competition of the two species; a positive CI value indicates a competitive advantage for the species on the numerator; a negative CI value indicates a competitive advantage for the species on the denominator. The Relative Increase Ration (RIR) was calculated based on the counts of viable cells [Log_10_(CFU/cm^2^)] obtained from single-species biofilms of each strain^12^.

### Confocal laser scanning microscopy (CLSM)

Single- and dual-species biofilms were formed on polystyrene coupons (NuncThermanox, Thermo Scientific, MA, USA) placed on 24-well polystyrene microtiter plates as described above, with slight modifications. The volume of bacterial suspension was adjusted to 1 mL/well instead of 200 μL/well. Expression of mCherry fluorophore and sfGFP was induced by adding 1 mM IPTG and 0.2% (v/v) of L-arabinose^54,55^, after 43 h of biofilms formation. Induction occurred during 5 h in the 48 h-old biofilms. Z-stacks were acquired on a CLSM (Olympus BX61, Model FluoView 1000, Olympus, Tokyo, Japan) equipped with 405–635 nm laser lines. Images were obtained with the FV10-Ver4.1.1.5 program (Olympus, Tokyo, Japan).

### Extraction of EPS from biofilms

*E. coli* and *S*. Enteritidis single- and dual-species biofilms were grown using the colony biofilm procedure^56^, with some modifications. Polycarbonate sterile membrane filters (Whatman®, Maidstone, UK, 47 mm diameter, 0.2 μm pore size) were placed on solid LB plates with the shiny side facing up. Each membrane was inoculated with 50 µL of EC 434, EC 515, SE Ex2, SE 269, or alternatively with the mixtures of EC 434 + SE Ex2 or EC 515 + SE 269. All strains were diluted from overnight cultures to a final concentration of ≈1 × 10^8^ CFU/mL. Biofilms were formed for 48 h at 37 °C without agitation, and membranes were transferred to fresh LB plates every 24 h. For each strain or combination of strains, ten membranes were used. The extraction of EPS from biofilms was performed as previously described^57^.

### Fourier transform infrared-attenuated total reflectance (FTIR-ATR) spectroscopy of EPS

EPS of biofilms was lyophilized and analyzed by FTIR-ATR spectroscopy. Samples were transferred to the ATR crystal and a pressure of 150 N/cm^2^ was applied. Infrared spectra were acquired (PerkinElmer Spectrum BX FTIR System spectrophotometer, PerkinElmer, Waltham, USA) with a PIKE Technologies Gladi ATR accessory (PIKE Technologies, Inc., Madison, USA) from 4000 to 600 cm^−1^ with a resolution of 4 cm^−1^ and 32 scans co-additions. For each sample, three instrumental replicates (obtained in the same day) and two biological replicates (obtained in two different days from two independent bacterial growths) were obtained, corresponding to a total of six spectra for each extracted EPS. Between each EPS measurement, the background was acquired. Spectra corresponding to the instrumental replicates were averaged prior to the analysis.

### Phage propagation and titration

SE Ex2 and EC 434 were used for the production and titration of phages φ135 and Daica. Phages were amplified using the plate lysis and elution method^58^. Titration of the phage was performed according to a previously described protocol^59^.

### Transmission electron microscopy (TEM) analysis of phages

The morphology of phage particles was observed by TEM as previously described^60^.

### Phage DNA extraction, genome sequencing, and annotation

Phage DNA was extracted as described before^60^. Phage DNA was prepared for sequencing using the Illumina Nextera XT library preparation kit generating 250 bp paired-end sequencing reads and sequenced in the Illumina HiSeq platform. Reads were trimmed to remove adapters, contaminations, or low-quality sequences, and assembled with the CLC Genomics Workbench version 7 (CLC Bio, Aarhus, Denmark). Structural annotation was conducted with MyRAST^61^ and also checked manually (Geneious 9.1.4, Biomatters, NJ, USA). Potential frameshifts were checked with BLASTX^62^. Gene functions were predicted using BLASTP programs^63^ (E-value ≤ 10^−5^) and HHPRED server^64^, consulted between June and July 2018, TMHMM^65^ and Phobius^66^. The search of tRNA encoding genes was performed using tRNAscan-SE^67^. Putative promoters were searched using PhiSITE^68^, and putative regions were manually verified. ARNold^69^ was used to predict Rho-independent terminators and the energy was calculated using Mfold^70^. Protein parameters were determined using Sequence Manipulation Suite: Protein Isoelectric Point and Protein Molecular Weight^71^. Whole-genome comparisons were performed using EasyFig^72^ and OrthoVenn^73^. The complete genome sequences of Daica and phage φ135 were deposited in GenBank under accession nos. MH992509 and MH992510.

### Phage one-step growth characteristics

The one-step growth curves of phages Daica and φ135 on the two tested *E. coli* and *S*. Enteritidis strains were performed as described previously^51^ using a multiplicity of infection (MOI) of 0.001. Experiments were performed in triplicate.

### Biofilm treatment with phages

Biofilms formed on 96-well microtiter plates for 24 h, as described above, were washed once with saline, and exposed to 100 μL of LB and 100 μL of phage at a multiplicity of infection (MOI) of 1. Single-species biofilms of *S*. Enteritidis were exposed to φ135, and *E. coli* to Daica. Dual-species biofilms were exposed to both phages. Microtiter plates were incubated at 37 °C, 120 rpm, and samples were taken after 4, 8 and 24 h. Control experiments were performed using 100 μL of LB and 100 μL of SM buffer (5.8 g/L NaCl, 2 g/L MgSO_4_.7 H_2_O, 50 mL 1 M Tris, pH 7.5). The number of viable biofilm cells (CFU/cm^2^) was determined before and after phage infection, with serial dilutions performed in saline-FAS solution [0.9% (w/v) NaCl, 2 mM of ferric ammonium sulfate (FAS)] to destroy non-infecting phages^74^. Experiments were performed in triplicate.

### Infrared spectra data analysis

Two principal component analysis (PCA) models^75^ were developed including EPS spectra from the two *E. coli* and two *S*. Enteritidis strains, in single- and dual-species biofilms. Prior modeling, spectra were pre-processed with standard normal variate (SNV) followed by the application of a Savitzky-Golay filter (15 smoothing points, 2^nd^ order polynomial, and 1^st^ derivative)^76,77^ and mean-centered. All data analyses were performed in Matlab version 7.9 (Mathworks, USA) and the PLS Toolbox version 5.5.1 for Matlab (Eigenvector Research, USA).

### Statistical analysis

Statistical analysis of the results was performed using GraphPad Prism 6 (GraphPad Software, CA, USA). The independent experiments and the results are presented as mean ± standard deviation (SD). Differences between single- and dual-species biofilm formation abilities were assessed using two-way ANOVA followed by Tukey’s multiple comparison statistical test. CI and RIR were compared using unpaired Student’s *t*-test, with significant differences being indicative of a probable competition between the species^12^. Differences were considered statistically significant if p < 0.05 (95% confidence interval).
