# Author Correction: *Escherichia coli* and *Salmonella* Enteritidis dual-species biofilms: interspecies interactions and antibiofilm efficacy of phages

**DOI:** 10.1038/s41598-020-59306-7

**Published:** 2020-02-06

**Authors:** Catarina Milho, Maria Daniela Silva, Diana Alves, Hugo Oliveira, Clara Sousa, Lorenzo M. Pastrana, Joana Azeredo, Sanna Sillankorva

**Affiliations:** 1 Centre of Biological Engineering, LIBRO – Laboratório de Investigação em Biofilmes Rosário Oliveira, University of Minho, 4710-057 Braga, Portugal; 2 LAQV/REQUIMTE, Chemical Science Department, Faculty of Pharmacy, University of Porto, 4050-313 Porto, Portugal; 3 INL- International Iberian Nanotechnology Laboratory, Av. Mestre José Veiga, 4715-330 Braga, Portugal

Correction to: *Scientific Reports* 10.1038/s41598-019-54847-y, published online 03 December 2019

In Figure 8, the key is missing. The correct Figure 8 appears below as Fig. 1.

**Figure 1:**
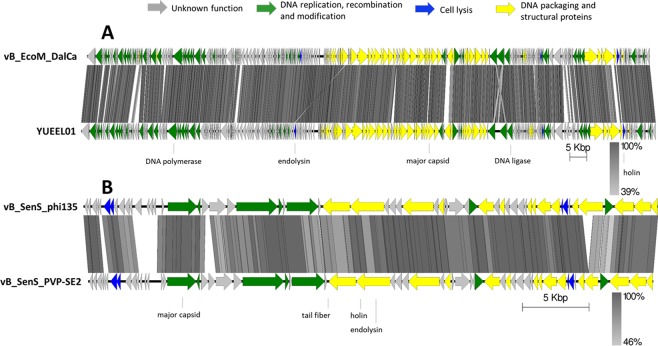
.

